# Effectiveness of preoperative micronized purified flavonoid fraction treatment and sucralfate-based rectal ointment on hemorrhoidal disease: A case-matched analysis

**DOI:** 10.1007/s10151-024-02998-0

**Published:** 2024-09-17

**Authors:** G. Gallo, E. Lori, M. Goglia, A. Dezi, A. Picciariello, U. Grossi

**Affiliations:** 1 Department of Surgery, Sapienza University of Rome, Rome, Italy; 2 Ph.D. School in Translational Medicine and Oncology, Department of Medical and Surgical Sciences and Translational Medicine, Faculty of Medicine and Psychology, Sapienza University of Rome, Rome, Italy; 3 Department of Precision and Regenerative Medicine and Ionian Area, University Aldo Moro of Bari, Bari, Italy; 4 Department of Experimental Medicine, University of Salento, Lecce, Italy; 5 Department of Surgery, Oncology and Gastroenterology—DiSCOG, University of Padua, Padua, Italy

**Keywords:** Hemorrhoidal disease, Preoperative treatment, Flavonoids, Sucralfate, Rectal ointment, Symptom severity

## Abstract

**Background:**

Hemorrhoidal disease (HD) significantly impacts patients’ quality of life. This study aimed to evaluate the effectiveness of preoperative treatment with the micronized purified flavonoid fraction (MPFF) and a sucralfate-based rectal ointment in managing HD symptoms and reducing interventions.

**Methods:**

A prospective quasi-experimental study including consecutive cases and controls matched on the basis of sex was performed in a tertiary referral center. Cases received systemic and local therapy for HD, consisting of a rectal ointment containing 3% sucralfate and herbal extracts plus MPFF, in addition to conservative therapy, while controls received conservative therapy alone. The hemorrhoidal disease symptom score (HDSS), the Short Health Scale for HD (SHS-HD) score, and the Vaizey Incontinence Score were used to evaluate symptoms severity and their impact on quality of life and continence. Intervention requirements were assessed at baseline (T0) and after 60 days of treatment (T1).

**Results:**

Between January and December 2023, a total of 98 patients were assessed for eligibility. After exclusions, 56 patients were enrolled, with 28 in each group. Significant improvements were observed in HD symptom scores from T0 to T1: the intervention group showed a mean change in HDSS of −9 [95% confidence interval (CI) −10 to −8], and the control group showed no significant change (mean change of 0; 95% CI −1.5 to 0). At T1, a higher proportion of patients in the intervention group underwent less invasive interventions compared with controls (18% versus 11%). Age, treatment group, and baseline symptom severity significantly predicted post-treatment symptom scores.

**Conclusions:**

In our study the preoperative treatment with MPFF and a sucralfate-based rectal ointment demonstrated clinical benefits in managing HD symptoms and reducing interventions. Further prospective trials are warranted to confirm and explore additional therapeutic strategies.

**Supplementary Information:**

The online version contains supplementary material available at 10.1007/s10151-024-02998-0.

## Introduction

Hemorrhoidal disease (HD) is a prevalent benign yet distressing condition, affecting approximately 39% of the general population at least once in their lifetime and significantly impacting their quality of life [1]. Despite its common occurrence, there is often a discordance between the severity of HD and its symptomatic manifestation [2].

Traditional classification systems, such as the widely recognized Goligher classification, have been employed to categorize the disease’s severity on the basis of the presence and degree of prolapse [3, 4]. However, this classification has limitations, particularly in its minimal consideration of symptoms such as bleeding in Grade I HD and the extent of inflammatory involvement [5], [6].

Recent advancements have introduced various conservative therapies aimed at either serving as definitive treatments or preparing patients for surgery. Oral phlebotonics, in particular, have shown efficacy in reducing persistent symptoms—such as bleeding, itching, and pain—and in preventing recurrence of HD [7].

These therapies include a diverse array of medications derived from natural sources, such as flavonoids, or synthesized compounds, such as calcium dobesilate [8]. Initially recognized for their use in early and less severe cases or those unsuitable for surgery, these treatments are now also employed for downstaging the disease pre-surgery and occasionally as definitive treatments.

The present study examines the efficacy of two such products currently available on the market: a rectal ointment containing 3% sucralfate and herbal extracts (calendula, witch hazel leaf, and chamomile), branded as Emoflon™, and micronized purified flavonoid fraction (MPFF), a combination of 90% diosmin and 10% other flavonoids, known as Daflon® [9, 10]. The rectal ointment aims to establish a barrier effect, mitigate irritative stimuli, reduce trauma from stool transit, and minimize microbial exposure to the anorectal region [11, 12].

This study aims to assess the changes in symptom severity and intervention requirements following treatment with MPFF and a topical rectal ointment, in comparison to a matched control group.

## Methods

### Study design

The present study was developed in accordance with the Strengthening the Reporting of Observational Studies in Epidemiology (STROBE) checklist (Appendix 1) [13].

This study was designed to assess the effectiveness of a new treatment regimen for HD compared with standard care. It employs a quasi-experimental design, which is characterized by the comparison of an intervention group and a control group without the random assignment of participants to these groups. Quasi-experimental designs are commonly used in situations where randomization is not feasible or ethical, allowing for the examination of causal relationships in real-world settings [14].

### Enrollment timeline

- Controls: patients who received standard care were consecutively enrolled between January and June 2023.
- Cases: patients who received the new treatment regimen were consecutively enrolled between September and December 2023.

This staggered enrollment was planned to allow for an initial assessment of the standard care outcomes before introducing the new treatment regimen. This design helps establish a historical control group for comparison with the intervention group. Controls were prospectively enrolled during the first semester of 2023, receiving standard conservative management before the new treatment protocol was initiated in the second semester. This ensured that control group patients had not been exposed to the specific treatments under evaluation.

### Treatment groups

- Controls: they received pain relief therapy as needed (no painkillers needed; minor or major analgesics), lifestyle modifications, fiber supplementation, and stool softeners [15].
- Cases: they received systemic and local therapy for HD in addition to the control therapy. The additional therapy included:
-
  - Rectal ointment twice daily: patients received the rectal ointment containing 3% sucralfate and herbal extracts (Emoflon™, manufactured by Egis Pharmaceuticals PLC). This medication is available in Italy from 2019 and is intended for the treatment HD symptoms and postoperative complications [16].
    - MPFF: additionally, patients in the experimental group were administered a 500 mg MPFF twice daily. MPFF consists of 90% micronized diosmin and 10% other flavonoids (hesperidin, diosmetin, linarin, and isorhoifolin). This compound improves venous tone and lymphatic drainage, reducing capillary hyperpermeability and protecting the microcirculation from inflammatory processes. Indeed, MPFF has the ability to inhibit leukocyte–endothelium interaction, thus preventing the activation of the inflammatory cascade involving cytokines, prostaglandins, leukotrienes, histamine, and other inflammatory mediators. Diosmin’s absorption is enhanced by micronization to particles less than 2 microns [17].

### Intervention initiation and preoperative assessment

The study consisted of a baseline time (T0) of patient’s first assessment and a follow-up visit at T1, after 60 days of conservative treatment, which corresponded to 7 days before the secondary procedure.

At T0, a spectrum of interventions, ranging from conservative to surgical [i.e., sclerotherapy, hemorrhoidal artery ligation-recto anal repair (HAL-RAR), or hemorrhoidectomy], were discussed with the patient on the basis of symptomatic presentation, prior consultations, and current guidelines [8, 18]. To minimize bias in surgical decision-making, reassessment at T1 was conducted by an independent surgeon not involved in the initial patient assessment (T0). Despite this, we acknowledge that the decision to alter the surgical plan remains subjective and can introduce bias. The reassessment had to evaluate whether the patient became eligible for less invasive alternative procedures due to improved outcomes, or eventually whether he needed escalation toward more invasive measures. All choices were discussed and made on the basis of patient response and preferences.

Demographic data, degree of symptoms of HD, quality of life, and continence level were collected through prospective data collection. The scale and type of analgesic intake were documented at T0 and compared between the two groups. Pain was assessed with a visual analogue scale (VAS) score (minimum score = 0, maximum score = 10) as well as the amount (0 = no painkillers needed; 1 = 1–3 per day for up to 2 days; 2 = 1–3 per day for more than 2 days; 3 = more than 3 per day for more than 2 days) and type of painkillers (no painkillers needed; minor analgesics: 1000 mg paracetamol; major analgesics: 600 mg ibuprofen or 1000 mg paracetamol with 60 mg codeine) [19].

### Eligibility criteria

Patients aged between 18 and 75 years with symptomatic HD according to the Goligher classification were eligible for the study.

Patients with a history of cardiac disease, coagulopathy and anticoagulant therapies, colorectal or anal neoplasia, inflammatory bowel disease, other proctological diseases (anal fistulas and fissures, thrombosed internal or external hemorrhoids), previous anal surgical procedures, previous sclerotherapy or rubber band ligation in the last 12 months, positive pregnancy test or breastfeeding, hepatitis B virus, hepatitis C virus, or human immunodeficiency virus infection, proctitis, known allergy to polidocanol, pelvic radiotherapy, or inability to return for postoperative follow-up visits were excluded from the study.

Written informed consent was obtained from all patients included in the study.

### Procedure (treatment plan)

All patients underwent a proctological examination including digital rectal examination and anoscopy to confirm the severity of HD and rule out associated anorectal diseases. Treatment including sclerotherapy, HAL-RAR, or hemorrhoidectomy was re-determined at T1 on the basis of the response to therapy initiated at T0.

### Outcomes

The primary outcome was the modification in symptom severity from T0 to T1.

The severity of symptoms was assessed using validated scores, including the Hemorrhoidal Disease Symptom Score (HDSS) and Short Health Scale HD (SHS-HD) [20] and Vaizey incontinence score [21] at both T0 and T1.

Secondary outcomes included the rate of downgrading in intervention type between T0 and T1, measured as the percentage of patients initially recommended for a more invasive intervention (e.g., hemorrhoidectomy) at T0 who were advised a less invasive or no intervention at T1 instead.

### Statistical analysis

Descriptive statistics were used to summarize the demographic and clinical characteristics of the study population. Categorical variables were reported as frequencies and percentages, while continuous variables were reported as means with standard deviations or medians with interquartile ranges, as appropriate. Inferential statistics were used to compare outcomes between the control and experimental groups. A linear regression analysis was performed to assess the impact of treatment group (cases versus controls), age, and baseline HDSS at T0 on the post-treatment HDSS at T1. Statistical significance was set at a *p*-value of less than 0.05.

## Results

**Fig. 1:**
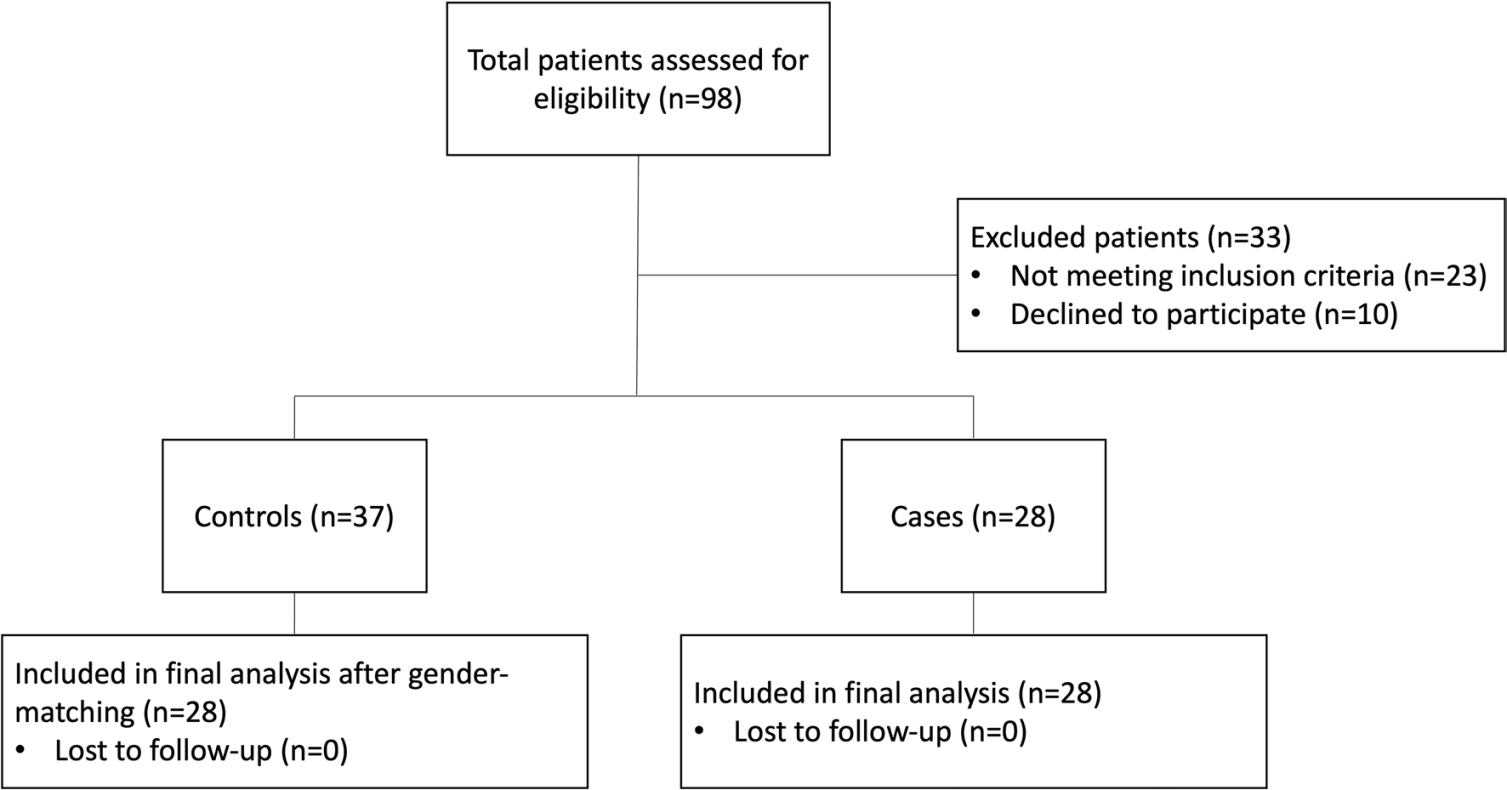
Patient flowchart

During the study period, a total of 98 patients was initially assessed for eligibility (Fig. 1). After applying the inclusion and exclusion criteria, 33 patients were excluded for various reasons: 23 did not meet the inclusion criteria, and 10 declined to participate. From the enrolled patients, the 28 cases were sex-matched with 28 controls selected from the pool of 37 controls. Consequently, a total of 56 patients was enrolled, with a distribution of 34 (61%) males and 22 (39%) females. The median age was 52 years (range, 18–74). Baseline characteristics, including age, Goligher grade, and symptom severity, remained consistent between the groups, as evidenced by the planned therapeutic indications at T0 (Table 1). Only a minority of them were deemed suitable for an office-based procedure, i.e., sclerotherapy (18% in both groups).

**Table 1:** Patients’ characteristics

	Groups		*p*-Value
	Intervention (*N* = 28)	Control (4 = 28)	
Sex (*N*, %)			
Female	11 (39)	11 (39)	/
Male	17 (61)	17 (61)	
Age (median, IQR)	45 (29–59)	52 (48–57)	0.187
Goligher grade (*N*, %)			
II	7 (25)	5 (18)	0.704
III	16 (57)	19 (68)	
IV	5 (18)	4 (14)	
Type of analgesia (*N*, %)			
None	11 (39)	8 (29)	0.080
Minor	8 (29)	16 (57)	
Major	9 (32)	4 (14)	
Frequency of analgesia (*N*, %)			
None	11 (39)	7 (25)	0.01
1–3 daily for ≤ 2 days	9 (32)	19 (68)	
1–3 daily for > 2 days	8 (29)	2 (7)	
HDSS (median, IQR)	10.5 (9–12)	10 (9–11)	0.495
SHS (median, IQR)	16 (14–20)	18 (14–20)	0.347
Vaizey score (median, IQR)	0 (0–1)	0 (0–1)	0.17
Planned treatment (*N*, %)			
Sclerotherapy	5 (18)	5 (18)	0.957
HAL-RAR	13 (46)	12 (43)	
Hemorrhoidectomy	10 (36)	11 (39)	

*IQR* interquartile range, *HDSS* Hemorrhoidal Disease Symptom Score, *SHS* Short Health Scale, *HAL-RAR* hemorrhoidal artery ligation-recto anal repair

### Outcomes

**Fig. 2:**
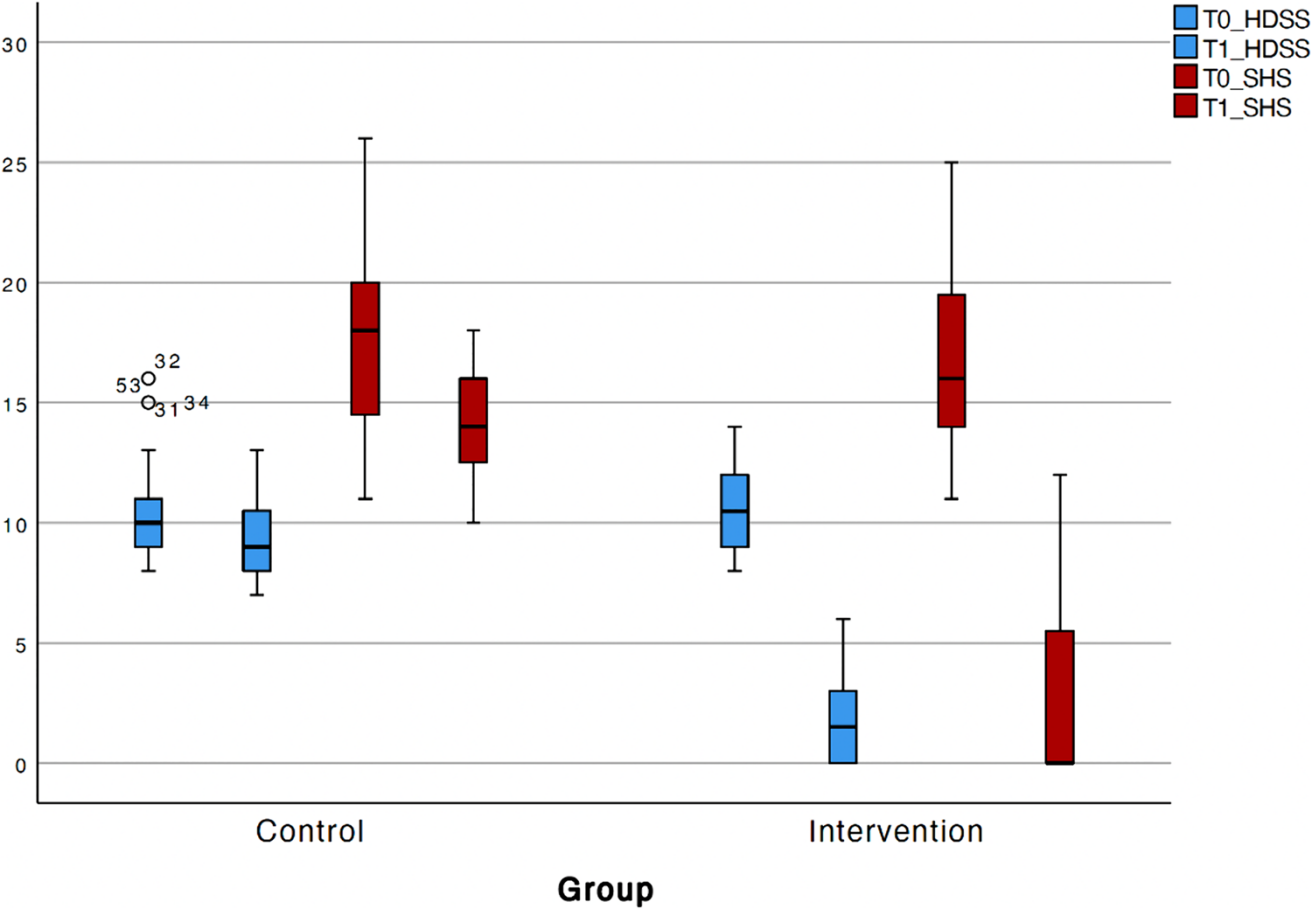
Variations in HDSS and SHS-HD from T0 to T1 between groups (mean, 95%CI)

A statistically significant improvement (*p* < 0.001) in HDSS and SHS-HD was observed from T0 to T1. A greater reduction in HDSS was noted in the intervention group compared with the control group. Specifically, the mean change in HDSS from T0 to T1 was −9 (95% CI −10 to −8) in the intervention group, whereas there was no significant change in the control group (mean change of 0, 95% CI −1.5 to 0; *p* < 0.001) (Fig. 2). Similar results were observed for SHS-HD, with a mean change of −14 (95% CI −16 to —10) in the intervention group compared with −1 (95% CI −6 to 0) in the control group. The Vaizey score remained unchanged during the study period. No adverse events occurred in both groups.

In the linear regression model predicting post-treatment HDSS at T1, the analysis revealed that age was not a statistically significant predictor (*p* = 0.578). However, both the treatment group (*p* < 0.001) and baseline HDSS at T0 (*p* = 0.011) were significant predictors of the post-treatment HDSS at T1. Specifically, treatment in the intervention group, compared with controls, was associated with a lower HDSS at T1, suggesting an improvement in symptoms. Additionally, the coefficient for baseline HDSS at T0 was 0.141 (95% CI 0.071–0.515), indicating that higher symptom levels at baseline were associated with greater symptom severity at T1, regardless of the treatment received.

**Fig. 3:**
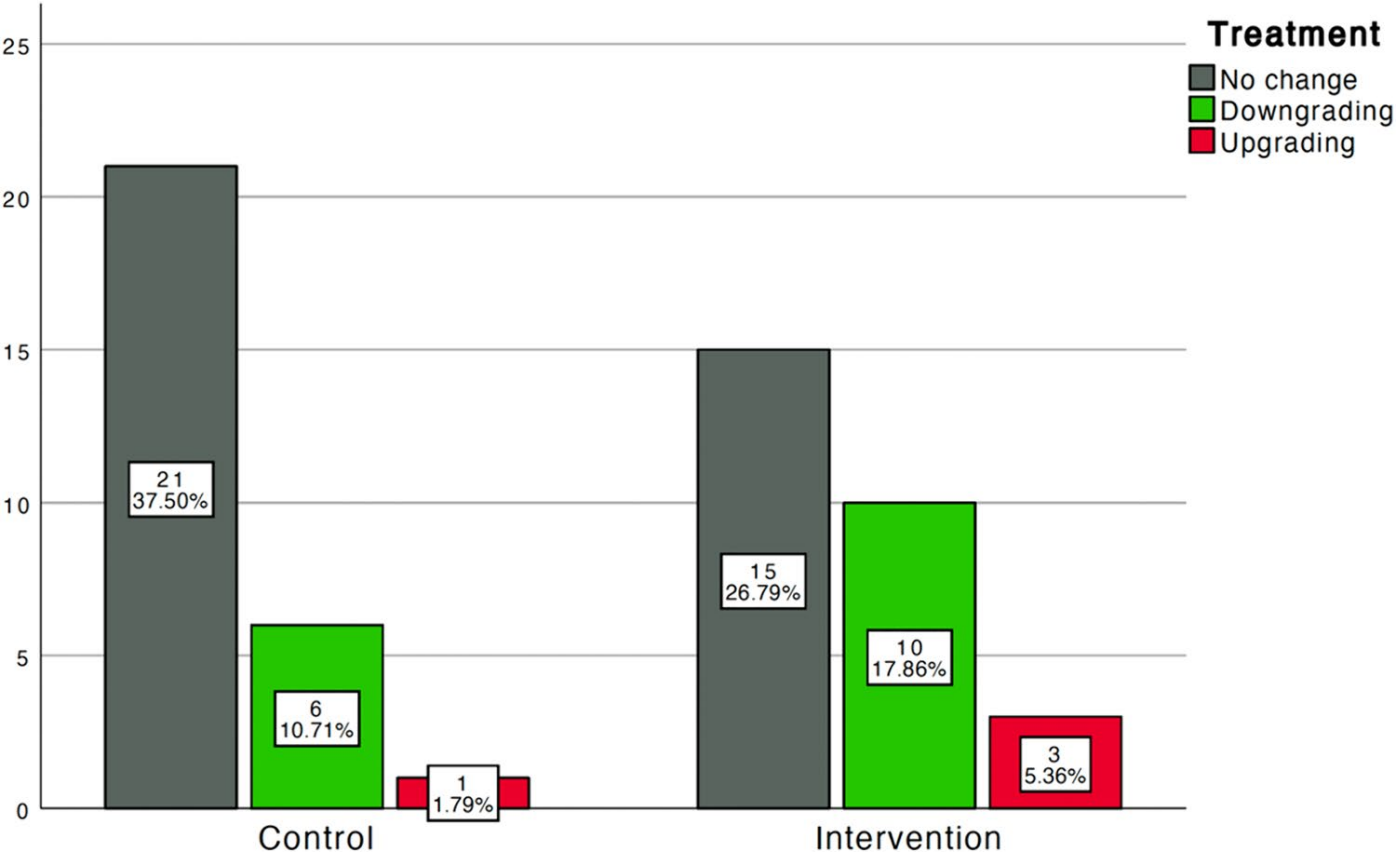
Distribution of patients receiving the same treatment, a less invasive, or a more invasive treatment from T0 to T1 between groups

Compared with the control group, a higher proportion of patients in the intervention group received less invasive treatment from T0 to T1 (10 [18%] versus 6 [11%]; Fig. 3). The planned treatment at T0 aligned with the actual treatment at T1 for 36 (64%) patients (Table 2). Only four patients underwent a more invasive treatment, with three in the intervention group (from HAL-RAR to hemorrhoidectomy [*n* = 2] or from sclerotherapy to HAL-RAR [*n* = 1]) and one in the control group (from sclerotherapy to HAL-RAR).

**Table 2:** Distribution of treatments (planned and actual) among groups

Treatment		Groups (*N*, %)		
Planned	Actual	Intervention	Control	
Sclerotherapy	Sclerotherapy	3 (11)	4 (14)	**No change ** ***N* = 36 (64%)**
HAL-RAR	HAL-RAR	5 (18)	9 (32)	
Hemorrhoidectomy	Hemorrhoidectomy	7 (25)	8 (29)	
Sclerotherapy	No treatment	1 (4)	0	**Downgrading** ***N* = 16 (29%)**
HAL-RAR	Sclerotherapy	6 (21)	3 (11)	
Hemorrhoidectomy	HAL-RAR	3 (11)	3 (11)	
Hemorrhoidectomy	Sclerotherapy	0	0	
Sclerotherapy	HAL-RAR	1 (3)	1 (3)	**Upgrading** ***N* = 4 (7%)**
Sclerotherapy	Hemorrhoidectomy	0	0	
HAL-RAR	Hemorrhoidectomy	2 (7)	0	

*HAL-RAR* hemorrhoidal artery ligation-recto anal repair

## Discussion

This prospective quasi-experimental study underscores the effectiveness of preoperative treatment for HD. Specifically, the combination of a rectal ointment and MPFF resulted in a significant reduction in the treatment group’s score compared with the control group, alongside a concurrent downstaging of HD and the subsequent selection of less invasive treatments (11% versus 18%). Notably, several benefits were also observed in the control group, indicating the effectiveness of non-medical conservative strategies such as increased fiber and water intake, stool softeners, and local hygiene measures. It is noteworthy that only one patient in the control group experienced worsening of the intervention type.

The efficacy of oral flavonoids across all HD stages has been extensively documented. A recent meta-analysis incorporating data from 22 randomized controlled trials (RCTs) and 2335 patients evaluated the efficacy and safety of MPFFs in treating postoperative hemorrhoid complications, demonstrating their role in reducing bleeding rates, pain, and edema scores, as well as enhancing the clinical efficacy of hemorrhoidectomy without adverse reactions [22]. These findings were further supported by Medjova et al. [23], who reported a significantly lower rate of thrombosis or edema of mucocutaneous bridges after excisional hemorrhoidectomy, along with higher patient-assessed treatment effects in comparison to the control group.

Alonso-Coello et al. were among the first to highlight the beneficial effects of flavonoids on acute HD symptoms [7]. Although optimal pre- and post-operative dosages and durations of medication remain uncertain, the authors suggested that high-dose MPFF (i.e., 3000 mg per day for the first 4 days and 2000 mg per day for 3 consecutive days) is most effective in the acute phase, while dosage reduction can be considered during the relief phase (i.e., 1000 mg per day for 2 months), depending on symptoms. High dosage efficacy was also supported by Fu et al. [22]. In particular, 1800–2700 mg per day of MPFF was able to significantly reduce edema and pain scores, while less than 1800 mg per day demonstrated significant improvement in the clinical efficacy of the excisional procedure.

Our study maintained a constant dosage of 1000 mg per day, supplemented with topical ointment application to augment therapeutic effects, warranting further investigation through future randomized trials.

Limited research has explored the role of combined local and systemic therapies for HD. Amaturo et al. [12] reported significant symptom reduction and high patient satisfaction scores following a combination of oral MPFF and topical sucralfate-based rectal ointment. Similarly, Gupta et al. [16] demonstrated the effectiveness of sucralfate cream over placebo in patients undergoing excisional hemorrhoidectomy, attributing its efficacy to higher bioavailability and minimal systemic side effects.

Despite its contributions, this study has limitations, including its non-randomized design and small sample size. However, the quasi-experimental approach, coupled with statistical matching, mitigates some randomization biases. The control group did not receive any specific product aside from basic conservative therapy. This decision was motivated by the patients’ absolute choice to undergo surgery (either non-excisional or excisional) due to previous unsuccessful medical treatment. Moreover, the efficacy of this conservative approach has been extensively supported by national and international guidelines [8, 18]. While there is a potential for selection bias, controls were chosen prospectively and had not failed the intervention treatment. They were managed conservatively during the first semester of 2023 before the new treatment protocol began, minimizing bias and ensuring comparability between groups. However, it is worth considering that, within the same classification grade (e.g., Goligher II degree), patients may have had options for different interventions, such as HAL-RAR or sclerotherapy, as dictated by guidelines and patient preferences, reflecting variations in recovery expectations and future recurrence risk. One significant limitation of our study is the subjective nature of altering surgical intervention plans on the basis of treatment response. Although an independent surgeon conducted the reassessment, the decision is inherently subjective. Future studies should consider implementing an independent review panel to further mitigate this bias. The study’s strengths lie in the use of validated scores for HD symptom evaluation and quality of life impact, alongside the involvement of two independent investigators throughout the study. Finally, the novel evaluation of preoperative patient assessment and strategy alteration could be a promising approach for HD management, potentially reducing healthcare costs.

## Conclusion

Our study underscores the clinical benefits of combined local and systemic therapy with MPFF and sucralfate-based rectal ointment in HD patients, promoting disease degree reduction and facilitating less invasive procedures in some cases. Prospective randomized clinical trials are needed to support these findings.

## Supplementary Information

Below is the link to the electronic supplementary material.

Supplementary file1 (PDF 593 KB)

## Data Availability

The data that support the findings of this study are available from the corresponding author upon reasonable request.
